# *In situ* observation of a macrourid fish at 7259 m in the Japan Trench: swimbladder buoyancy at extreme depth

**DOI:** 10.1242/jeb.246522

**Published:** 2024-02-01

**Authors:** Imants G. Priede, Alan J. Jamieson, Todd Bond, Hiroshi Kitazato

**Affiliations:** ^1^ School of Biological Sciences, University of Aberdeen, Tillydrone Avenue, Aberdeen AB24 2TZ, UK; ^2^ Minderoo-UWA Deep-Sea Research Centre, School of Biological Sciences and Oceans Institute, The University of Western Australia, 35 Stirling Highway, Perth, WA 6009, Australia; ^3^ Department of Marine Environmental Sciences, Tokyo University of Marine Science and Technology, 4-5-7 Konan, Minato-ku, Tokyo 108-8477, Japan; ^4^ Danish Center for Hadal Research, Satellite office at TUMSAT, Tokyo University of Marine Science and Technology, 4-5-7 Konan, Minato-ku, Tokyo 108-8477, Japan

**Keywords:** Deep-sea fish, Teleosts, Swimbladder, Buoyancy, Hydrostatic pressure, Physiology

## Abstract

A macrourid, *Coryphaenoides yaquinae* sp. inc., was observed to be attracted to bait and exhibiting normal foraging behaviour during a period of 80 min within view of a baited video camera on the sea floor at 7259 m – the deepest ever observation of a fish species with a swim bladder. The buoyancy provided by an oxygen-filled swim bladder at 74.4 MPa pressure was estimated to be 0.164 N, at a theoretical energy cost of 20 kJ, 200 times less than the cost of equivalent lipid buoyancy. During normal metabolism, 192 days would be required to fill the swimbladder. At these depths, oxygen is very incompressible, so changes in volume during ascent or descent are small. However, swimbladder function is crucially dependent on a very low rate of diffusion of oxygen across the swimbladder wall. The oxygen in the swimbladder could theoretically sustain aerobic metabolism for over 1 year but is unlikely to be used as a reserve.

## Teaser

**Highlighted Article:** The deepest ever observation of a fish with gas-filled buoyancy exhibiting normal behaviour shows that gas secretion and retention function at the extreme hydrostatic pressures of hadal ocean trenches.

## INTRODUCTION

Teleost fishes are unique amongst the diversity of organisms that use gas-filled structures for buoyancy in that they have evolved gas secretion glands (Berenbrink et al., 2005) that can inflate the swimbladder to a pressure equal to that of the surrounding water (Alexander, 1966). This has enabled them to colonise the deep sea (Priede and Froese, 2013) in contrast to cephalopods such as nautiloids (Kanie et al., 1980) and *Sepia* (Sherrard, 2000) whose rigid air-filled shells implode at modest depths, and other organisms whose buoyancy bladders are compressed during descent. Swimbladders are often lost in bathypelagic species but are retained in at least half of deep-sea demersal teleost species (Marshall, 1960). In these fishes, the gas in the swimbladder is predominantly oxygen (Scholander and Van Dam, 1953). The gas gland has a rete of blood vessels which acts as a counter-current multiplier of oxygen partial pressure. The length of the rete capillaries increases from 5 mm in species living at 200 m depth to over 25 mm in fish living at 3000 m depth (Marshall, 1972). The wall of the swimbladder is also invested with increasing quantities of guanine crystals (Lapennas and Schmidt-Nielsen, 1977) with depth (Ross and Gordon, 1978) that prevent the outward diffusion of gas.

There is uncertainty as to the maximum depth at which swimbladders can usefully function. Applying the Ideal Gas Law, Marshall (1960) calculated that at 7200 m depth, the density of oxygen becomes equal to that of sea water, therefore providing no buoyancy, and suggested the swimbladder may then be more important as a store of oxygen. However, at such high pressures, oxygen deviates from an ideal gas and does provide buoyancy, albeit considerably less than for shallow water species (Alexander, 1966; Priede, 2018). Nevertheless, the energy cost of inflating a swimbladder to such high pressures may be prohibitive. Also, there may be a limit to the maximum pressure at which oxygen can be secreted. Gerth and Hemmingsen (1982) suggest that the Root effect, the reduction in haemoglobin oxygen carrying capacity by lactic acid that enables gas secretion (Berenbrink et al., 2005), may cease to function at pressures above 40 bar (4 MPa). High partial pressures of oxygen are toxic to living tissues of fishes (D'Aoust, 1969) although gas gland cells may be protected by high concentrations of superoxide dismutase (Morris and Albright, 1981). Such defence systems against reactive oxygen species become particularly important in deep-sea fishes (Pelster, 2021).

Nielsen and Munk (1964) describe the swimbladder and gas gland of the cusk-eel (Ophidiidae), *Holcomycteronus profundissimus* retrieved from 7160 m depth in the Sunda Trench and this has been regarded as the maximum depth for functioning swimbladders (Pelster, 1997). As the net fished the entire water column between the sea floor and surface, the specimen may not have been caught at the maximum depth, although Nielsen and Munk (1964) advance cogent arguments supporting this depth record. Individuals caught at such extreme depths may have been accidentally displaced out of their normal milieu and might be moribund or functionally impaired.

Here, we present *in situ* observations of a macrourid (*Coryphaenoides* spp.) displaying normal activity at 7259 m depth in the Japan Trench: the deepest observation of a fish with a swimbladder. This is very close to the maximum depth, 7176 m, at which Ophidiids have also been observed *in situ* (Jamieson et al., 2021), suggesting that ca. 7200 m represents the maximum depth limit for fish with swimbladders. We calculate the buoyancy and its metabolic cost, and discuss function at these extreme depths.

## MATERIALS AND METHODS

In August and September 2022 during the DSSV *Pressure Drop* ‘Ring of Fire 2022’ expedition to the NW Pacific Ocean trenches around Japan, 31 baited camera lander deployments were made between the depths of 4913 and 8022 m along two transects across the axis of the Japan Trench (38°–39°N, 143.6°–145°E). The baited camera landers, known as *Skaff*, *Flere* and *Closp*, were deployed in free-fall using an expendable ballast weight, and surfaced using buoyancy following acoustically triggered jettisoning of the ballast. The mean (±s.d.) time spent on the bottom was 437±54 min. Video data were acquired by high-definition (HD) video cameras (IP Multi SeaCam 3105; Deep Sea Power and Light, San Diego, CA, USA) mounted horizontally with ∼400 g of mackerel (*Scomber* spp.) bait secured in the centre of the field of view, 1 m in front of the camera. Depth and temperature were recorded by conductivity, temperature and depth (CTD) probes (SBE 49 FastCAT, SeaBird Electronics, Bellevue, WA, USA). Depth was calculated from pressure based on the TEOS-10 thermodynamic properties of seawater (IOC et al., 2010) using the Gibbs-Seawater Oceanographic Toolbox (McDougall and Barker, 2011).

The data presented here are from the *Closp* baited camera that landed on the sea floor on the eastern, open ocean side of the Japan Trench (38.5505°N, 144.165°E) on 13 September 2022. The recorded pressure was 74.41±0.007 MPa, from which the depth was calculated as 7259 m, and the bottom temperature was 1.83±0.0002°C. The lander remained on the sea floor recording video for 8 h 9 min.

The species of fish that appeared during this deployment were one individual marcrourid *Coryphaenoides yaquinae* sp. inc. and 20–30 liparids *Pseudoliparis belyaevi* Andriashev & Pitruk 1993. Two species of macrourid occur in the Japan Trench region, *Coryphaenoides armatus* (Hector 1875) and *Coryphaenoides yaquinae* Iwamoto & Stein 1974, but they are indistinguishable in these images. As the latter predominates at greater depths (Endo and Okamura, 1992), we assign this individual as *C. yaquinae* sp. inc. (Jamieson et al., 2012; Horton et al., 2021). This work was undertaken with permission from the Japanese Ministry of Foreign Affairs and Ministry of Agriculture, Forestry and Fisheries, with permits (MoFA Note Verbale, no. AO877 and certificate for Catching Marine Animals and Plants no. 022) issued to H.K. and A.J.J.

## RESULTS AND DISCUSSION

### *In situ* observations

**Fig. 1.**
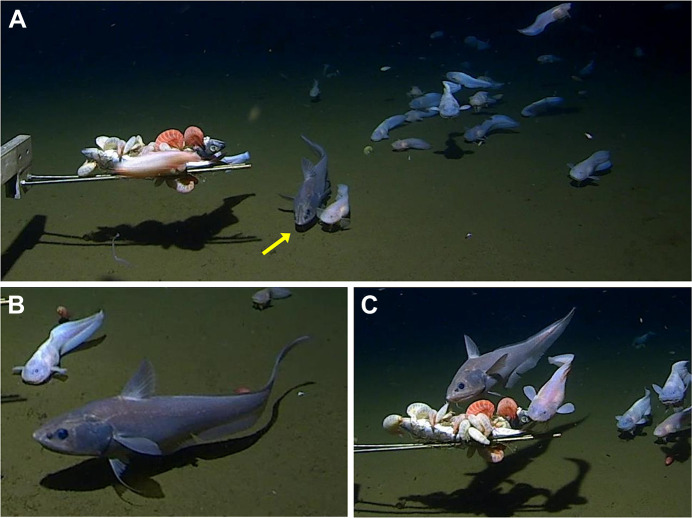
**A macrourid, *Coryphaenoides yaquinae* sp. inc., amongst liparids, *Pseudoliparis belyaevi*, attending bait at 7259 m depth in the Japan Trench.** (A) A macrourid (arrow) on the sea floor can be seen colliding with a snailfish (7 h 12 min after lander touchdown). The bait, supported on metal bars above the sea floor, is covered by amphipods (*Hirondella gigas*). (B) Lateral view of the macrourid 18 s later. (C) Feeding attempt at the bait at 7 h 13 min after lander touchdown.

Fishes arrived gradually until at 6 h 46 min after touchdown; 20 *P. belyaevi* were visible. Amphipods, predominantly *Hirondellea gigas* (Birstein & Vinogradov 1955), clustered on the bait and the fishes were manoeuvring in the odour plume downstream. A macrourid, *C. yaquinae* sp. inc., descended into the field of view amongst the liparids and departed after 45 s. The same presumed individual returned 10 times and was visible for 27% of the 80 min elapsed time before the lander departed from the sea floor (Movie 1). The macrourid swam amongst the liparids (Fig. 1A,B) and on two occasions ascended to feed on the bait about 25 cm above the sea floor (Fig. 1C). The mean tailbeat frequency was 0.68 Hz, varying between 0.74 and 0.61 Hz. We interpret the observations as a single persistent individual macrourid circling the bait source. In other lander deployments, up to 12 macrourids were observed from 4913 to 6000 m, with fewer at greater depths until only solitary individuals appeared at depths greater than 7000 m. The ophidiid *Bassozetus* sp. (morphotype 1: Jamieson et al., 2021) occurred between 5932 and 6882 m depth. *Pseudoliparis belyaevi* appeared in 23 of the 24 deepest deployments (6579–8022 m).

### How much buoyancy does the swimbladder provide?

**Fig. 2.**
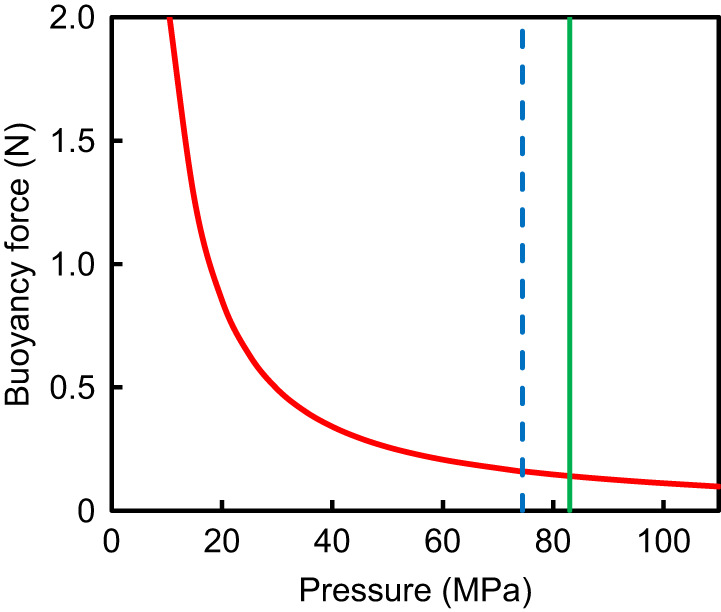
**Buoyancy force of a constant mass of 0.0369 kg of oxygen in seawater in relation to pressure.** The blue dashed line indicates 74.41 MPa, the depth at which this fish was observed. The green solid line indicates 83 MPa, the maximum depth limit for fishes.

At the recorded pressure of 74.41 MPa, the density of oxygen at 0°C is 739 kg m^−3^, interpolating from tables in Priede (2018). Assuming a fish of body mass 1 kg, within the mass range of *C. yaquinae* (Gerringer et al., 2018), and that 5% of the body volume is occupied by the swimbladder (0.05 l) (Martin et al., 2022), the mass of oxygen in the buoyancy organ is 0.0369 kg. In sea water, this would provide a buoyancy force of 0.158 N. Adjusting for the actual temperature, 1.83°C, the buoyancy increases slightly to 0.160 N but for convenience the calculations continue for 0.0°C. As the fish moves up and down in the water, the buoyancy would change as shown in Fig. 2. From 60 MPa pressure, at the margin of the trench, to 74.4 MPa, the buoyancy decreases from 0.206 to 0.158 N or a loss of 23% (Table S1). This is the same as that experienced by a surface-dwelling fish descending to 3 m.

### The swimbladder as a store of oxygen

Smith (1978) measured the oxygen consumption of *C. armatus*, *in situ* at 2750 and 3650 m and derived the relationship *Y*=0.03*M*^0.65^, where *Y* is the oxygen consumption in ml h^−1^ and *M* is the wet mass of the fish in g. This predicts a metabolic rate of 2.67 ml O_2_ h^−1^ or 3.82 mg O_2_ h^−1^. The amount of oxygen in the swimbladder is therefore equivalent to 0.0369×10^6^/3.82=9672 h or 1.1 years of normal oxygen consumption. The quantity of oxygen in the swimbladder could theoretically sustain the fish in hypoxic or anoxic conditions for long periods of time. However, hadal trenches are ventilated by cool deep water which is not anoxic (Priede, 2017) and high pressure reduces the solubility of oxygen (Enns et al., 1965), greatly increasing its partial pressure at hadal depths (MacDonald, 2021). As any oxygen from the swimbladder must enter the venous return to the heart, it would be lost to the surrounding water through the gills (Pelster, 2021) unless blood is shunted past the secondary lamellae (Hughes et al., 1982). It seems unlikely that macrourids use oxygen in the swimbladder as a reserve to sustain aerobic metabolism.

### Energy required to fill the swimbladder

**Fig. 3.**
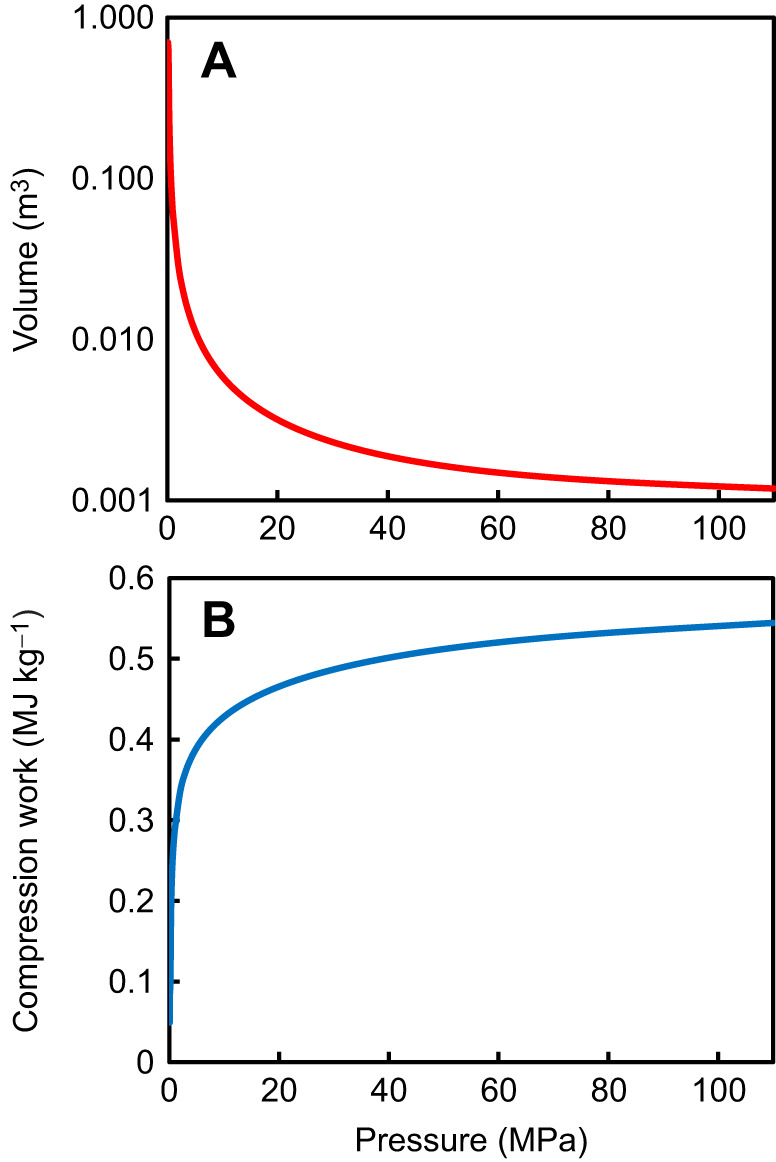
**Compression of 1 kg oxygen.** (A) Volume in relation to pressure. (B) Work done in compressing 1 kg of oxygen to the pressure indicated.

The work (*W*) done in isothermal compression of a gas from an initial state *a* to state *b* is given by the expression:
(1)

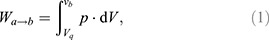
where *p* is the gas pressure and *V* is the gas volume. This can be calculated using the ideal gas law (*pV=nRT*) but as at high pressures oxygen does not behave as an ideal gas, an appropriate method is to integrate the area under the pressure–volume curve (Fig. 3).

Air is approximately 20% oxygen, so at atmospheric pressure the partial pressure of oxygen is 0.02 MPa. Over most of the deep Pacific Ocean, the dissolved oxygen is depleted; assuming approximately 50% of air saturation value, the partial pressure of dissolved oxygen can be assumed to be approximately 0.01 MPa. Hydrostatic pressure increases the effective partial pressure of dissolved gases (MacDonald, 2021); a pressure of 74.4 MPa would increase oxygen partial pressure to 0.033 MPa. Integrating numerically from 0.03 MPa to the ambient pressure at depth gives the relationship shown in Fig. 3B.

The work done in compressing oxygen to 74.4 MPa is 0.53 MJ kg^−1^ or 529 kJ kg^−1^. For the quantity of gas in the swimbladder, 0.0379 kg, the energy requirement is therefore 20.05 kJ. Assuming an oxycalorific value of 13.6 J mg^−1^ O_2_ (Elliott and Davison, 1975), this work would require consumption of 1475 mg of oxygen. At a metabolic rate of 3.82 mg O_2_ h^−1^, this is equivalent to 386 h or 16 days of metabolism. Assuming a lipid energy content of ca. 30 MJ kg^−1^, the gas secretion energy requirement is therefore equivalent to 0.67 g of lipid.

To attain the same buoyancy (0.164 N) using squalene would require 127 ml of oil with an energy value of 3.94 MJ (Priede et al., 2020) – orders of magnitude greater energy than that required to inflate the swimbladder.

### Efficiency and time required to fill the swimbladder

The work of swimbladder inflation is a combination of metabolic processes in the gas gland (Pelster, 2015) and the cost of pumping blood. The gas gland consumes glucose (Pelster and Scheid, 1993), which is largely metabolised anaerobically producing lactate that releases oxygen into the swimbladder (Pelster and Scheid, 1992). Kanwisher and Ebeling (1957) assumed the secretion process to be 25% efficient, expending no more than one-third of the metabolic rate. They give no reasons for these assumptions, but 25% corresponds to reported values for muscle contraction efficiency (Smith et al., 2005) and the one-third maximum can be inferred from metabolic scope considerations (Priede, 1985). Based on these values, the swimbladder can be filled over a period of 192 days, concurrent with all other metabolic activities. Experimental studies on shallow-water fishes give swimbladder filling times of 10–20 h (Blaxter and Tytler, 1978). At 74.41 MPa, the density of oxygen, 739 kg m^−3^, is 530 times greater than at the surface so gas secretion would be correspondingly longer: 221–440 days assuming similar gas gland performance. Over the likely 16 year life span of a 1 kg *C. yaquinae* (Gerringer et al., 2018), this is plausible assuming a gradual ontogenetic migration into deeper water, providing loss of gas from the swimbladder is minimal. However, some measurements of blood flow to the swim bladder (Pelster and Scheid, 1992) and cardiac output in eels, *Anguilla anguilla* (Claësson et al., 2016), suggest gas secretion rates may be an order of magnitude lower.

It is evident that the buoyancy cannot be adjusted rapidly, and these deep-living species probably maintain a constant mass of gas in the swimbladder. As oxygen is relatively incompressible at these high pressures, buoyancy change with descent from the abyssal plain into the trench is small (Table S1). Discovery of fish at 7259 m is not evidence of the capacity to secrete oxygen at such high pressures.

### Conclusion

The present observation of *C. yaquinae* sp. inc. at a depth of 7259 m represents a new maximum depth record for the family Macrouridae and indeed any fish with a swimbladder. It is close to the deepest confirmed sighting of a member of the family Ophidiidae, a *Bassozetus* morphotype at 7176 m depth in the Java Trench (Jamieson et al., 2021), and with the report by Nielsen and Munk (1964) of the Ophidiid *Holcomycteronus profundissimus* at 7160 m depth, thus removing the last vestiges of any doubt that fish with swimbladders can survive at depths greater than 7000 m. The fish was evidently close to neutral buoyancy, swimming slowly above the sea floor, and well coordinated, showing no signs of high-pressure pathology. The tail beat frequency was the same as observed in bait-attending macrourids (0.73 Hz) at 5800 m depth in the Central North Pacific (Priede et al., 2003). We conclude that Macrourids and Ophidiids can sustain normal function down to a depth limit of ca. 7200 m.

Here, we discount the report of an ophidiid *Abyssobrotula galatheae* at 8370 m (Nielsen, 1977), recently revised to 7965 m, as there has been no confirmation of occurrence at depths greater than 5875 m (Gerringer et al., 2021) despite multiple submersible visits to the same location and depth in the Puerto Rico Trench (Jamieson et al., 2020).

The only fishes at greater depths within hadal trenches are snailfishes, family Liparidae (Linley et al., 2016; Jamieson et al., 2021), which occur down to the theoretical maximum depth limit (8200–8400 m) for teleost fishes (Yancey et al., 2014). Liparidae do not possess a swimbladder and complete their entire life cycle within hadal trenches; this isolation leads to radiation into regional endemic species (Linley et al., 2022). Ophidiidae and Macrouridae produce buoyant eggs with larval development in the upper layers of the ocean (Priede, 2017).

We show that at 7259 m depth, an oxygen-filled swimbladder can provide significant buoyancy. Even the deepest-living macrourids therefore can maintain high bone density and strong jaws (Martin et al., 2022) aided by additional buoyancy from gelatinous tissue between the muscles of *C. yaquinae* (Gerringer et al., 2017). The ca. 7200 m depth limit of fish with swimbladders is unlikely to be related to a single physiological factor – rather, a combination of factors related to colonisation of the trench environment. Competitive exclusion by the numerically dominant liparids is possible. A key requirement for maintaining buoyancy and avoiding toxic effects of high-pressure oxygen is the impermeability of the swimbladder wall to gases. If gas escape is zero or negligible, swimbladders provide a very effective means of buoyancy at depth.

## Supplementary Material

10.1242/jexbio.246522_sup1 Supplementary information
