# Supplementary material for: In situ observation of a macrourid fish at 7259 m in the Japan Trench: swimbladder buoyancy at extreme depth
Source: J Exp Biol. 2024 Feb 1;227(3):jeb246522. doi: 10.1242/jeb.246522 (PMC10917060; doi:10.1242/jeb.246522)
Supplement: Supplementary information [file jexbio-227-246522-s1.pdf]

Table S1. The Effect of Pressure on density of Oxygen and Seawater, and the calculated swimbladder volume and buoyancy over the range 50-75 MPa at 0°C.

Pressure	Density			Swimbladder Volume 0.0369 kg of O_2		Buoyancy Force	
	O_2	Seawater	$\Delta\rho$				
MPa	kg.m^{-3}	kg.m^{-3}	kg.m^{-3}	m^3	ml	kg	N
50	614.48	1050.68	436.20	6.01303E-05	60.13	0.0262	0.257
55	644.87	1052.80	407.94	5.72968E-05	57.30	0.0234	0.229
60	672.11	1054.91	382.80	5.49746E-05	54.97	0.0210	0.206
65	696.10	1056.99	360.89	5.30796E-05	53.08	0.0192	0.188
70	718.42	1059.05	340.63	5.14308E-05	51.43	0.0175	0.172
74.41	738.97	1060.85	321.87	0.00005	50.00	0.0161	0.158
75	738.70	1061.09	322.39	5.00187E-05	50.02	0.0161	0.158

Oxygen density is from Priede (2018).

Seawater density is calculated from Fofonoff & Millard (1983) assuming salinity of 35 psu.

Δρ is the difference in density between oxygen and seawater (equal to buoyancy per m^3).

The grey shaded row is 7259 m depth at which the macrourid was observed.

References

Fofonoff, P. and Millard, R.C. Jr (1983) Algorithms for computation of fundamental properties of seawater. UNESCO Technical Papers in Marine Sciences 44, 53 pp.

Implemented by on-line calculator:

http://www.physocean.icm.csic.es/Utilities/calculators/density-en.html

Priede, I.G. (2018) Buoyancy of gas-filled bladders at great depth. *Deep Sea Res. Part I Oceanogr. Res. Pap*. **132,** 1-5. doi.org/10.1016/j.dsr.2018.01.004

Journal of Experimental Biology • Supplementary information

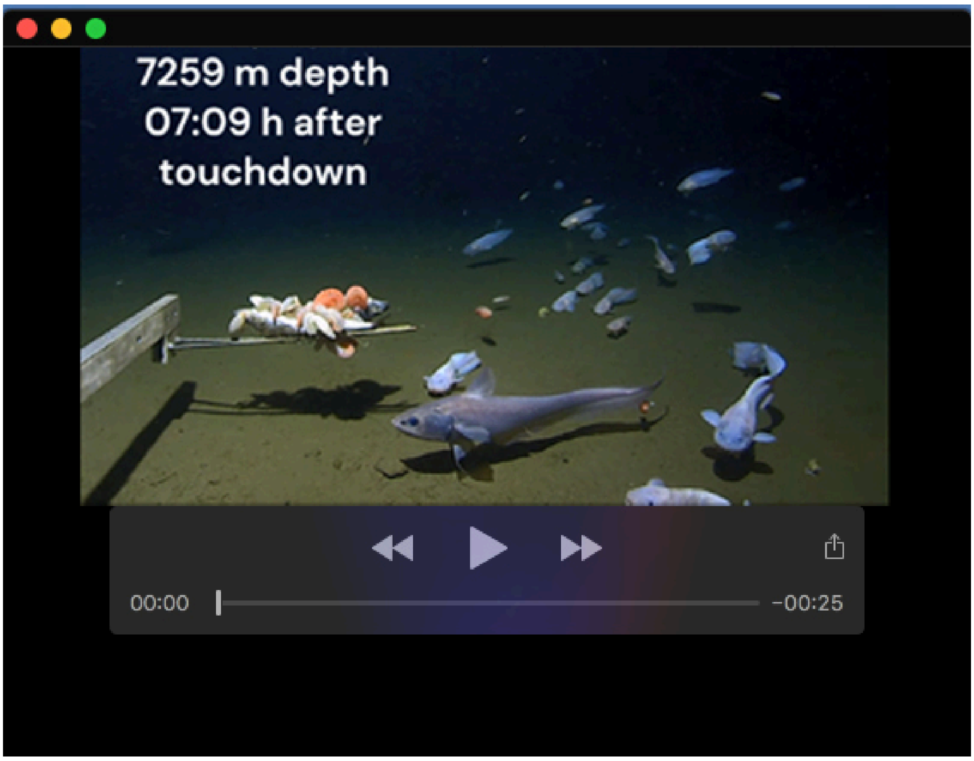

Movie 1. Lone macrourid approaching bait at 7259 m depth amongst a shoal of liparids.
